# Nurse Anesthetist-Performed PICC Insertion: A Prospective Longitudinal Study in a Norwegian Hospital

**DOI:** 10.1177/23779608251367258

**Published:** 2025-08-19

**Authors:** Ann-Chatrin Linqvist Leonardsen, Ellen Marie Lunde, Mona Sand Andersen

**Affiliations:** 1 Østfold Hospital Trust, Grålum, Norway; 2 Østfold University College/Østfold Hospital Trust, Halden, Norway; 3 University of Southeastern Norway, Kongsberg, Norway

**Keywords:** advanced practice nursing, complication, critical care outcomes, nurse anesthetists, observational study, peripherally inserted central catheter, venous access

## Abstract

**Introduction:**

The majority of hospitalized patients require vascular access for intravenous medical treatment. A peripherally inserted central catheter (PICC) may be indicated for long-term treatment. In many Norwegian hospitals, this is a nurse anesthetist-led initiative.

**Objective:**

The aim of this study was to examine the use of PICCs from insertion to completed treatment and to assess potential risk factors for the development of catheter-related complications.

**Methods:**

A quantitative prospective, longitudinal design was used. Data were collected via electronic patient records, as well as through telephone surveys with patients every fourth week until the catheter was discontinued. The Statistical Package for the Social Sciences version 28 was used for analysis.

**Results:**

In total, 401 PICCs were included. The primary indications were antibiotic treatment (*n* = 272), nutrition (*n* = 46), or chemotherapy (*n* = 42). A total of 163 PICCs were used for a period of over 30 days. Overall, 41 catheter-related complications were identified through patient records. Erythema at the insertion site (*n* = 12) was the most common complication, and the most severe complications were deep venous thrombosis (*n* = 5) and confirmed catheter-related infection (*n* = 4). After 4 weeks, 12 patients reported complications, with pain/numbness (*n* = 4) being the most frequent. The study demonstrated no statistically significant covariation between the occurrence of complications and age, length of time the catheter was in use, primary indication, venous diameter, or the number of insertion attempts.

**Conclusion:**

The study showed a low incidence of severe PICC-related complications. Peripherally inserted central catheters were inserted and used according to local guidelines. The study indicates that nurse anesthetists’ knowledge and experience can be utilized in the future selection and insertion of vascular access for patients, as well as in training and education on PICCs.

## Introduction

A majority of hospitalized patients require intravenous (IV) access for medical treatment such as drugs, fluids, nutrition, or blood (St Sauer et al., 2015). In addition, societal changes have led to a decentralization of healthcare services (Alper et al., 2017), creating a need for IV access in municipalities. Most often, IV access is obtained to provide therapies that cannot be administered or are less effective if given by alternative routes. Peripheral IV catheters have been the traditional choice, allowing for the safe infusion of medications, hydration fluids, blood products, and nutritional supplements (Frank, 2018). Traditionally, such catheters have been recommended for an interval of no more than three to four days (Young & You, 2025). Such short intervals challenge patients’ opportunity to receive IV treatment outside hospitals. Repeated discomfort due to insertion, as well as increased costs, complicate out-of-hospital IV treatment (Cheung et al., 2009). Hence, patients often need central venous access for long-term IV therapy. Central IV access devices are classified based on the duration of catheter use (short, medium, long), type of insertion (central, peripheral), location (jugular, brachial), number of lumens (single, double, triple), as well as whether the catheter is implanted (Chopra, 2019). Peripherally inserted central catheters (PICCs) are gaining in popularity due to the relative ease of insertion into the peripheral veins of the upper extremity and the ability to remain in place for one year or more (Biffi, 2014; Chopra, 2019; Hughes et al., 2014). In Norway, PICCs are commonly implemented and administered by nurse anesthetists (Smith & Haraldstad, 2019). They are inserted using ultrasound guidance, and placement is verified with either ECG or x-ray (Greca, 2014).

### Review of the Literature

Young and You (2025) found that long-term IV catheters are a significant source of catheter-related infections. Risk factors included diagnosis, catheter type, localization of the catheter, length of use, indication, observation, and the competence of the healthcare personnel involved. Furthermore, Paquet et al. (2017) found that using the right upper arm reduced the incidence of infections (23%) compared to using the left upper arm (34%). Additionally, Chopra (2019) indicated that risk factors included diagnosis, venous diameter, number of catheter lumens, arm used, and location of the catheter tip. The authors recommended that the catheter should be removed when not in use, a recommendation also supported by Young and You (2025), who recommended a dedicated venous access team to prevent complications. In line with this, Heffner and Androes (2024) emphasized the importance of informed decision-making and overall assessment of risks versus benefits related to choice of IV access. There is limited research into PICCs, as a central route to IV access, from the perspective of being initiated and administered by nurse anesthetists.

The aim of the study was to explore the insertion and use of PICCs in a Norwegian hospital where this has been initiated and implemented by nurse anesthetists.

## Methods

### Design

The study used a prospective, longitudinal, and observational design and followed The Strengthening the Reporting of Observational Studies in Epidemiology Statement (von Elm et al., 2007) (Supplement 1).

### Research Questions

The research questions were: (1) What were the characteristics of patients receiving a PICC during the study period? (2) What were the characteristics of the PICCs inserted? (3) What was the longitudinal incidence of complications? (4) Were there any covariations between complications and factors such as age, indication, venous diameter, number of venous punctures, and length of use?

### Sample

The study was conducted in a hospital with a catchment area of 320,000 inhabitants. In 2023, there were 1,062 PICCs inserted in the hospital. Here, PICCs are most commonly inserted in a dedicated room in the postoperative anesthesia care unit. Tip position is determined using ECG. In the hospital, 11 specially trained nurse anesthetists insert PICCs five days a week. The type of catheter is discussed with an anesthetist if in doubt. According to hospital guidelines, PICCs should only be used when the proposed treatment period extends over four weeks or in cases of abnormal pH/venous irritation of the fluid administered.

A purposeful sample strategy was used, including all PICCs inserted during the study period. Patients were recruited before PICC insertion by the nurse anesthetist performing the procedure. Information was provided both orally and written, and patients either gave oral or written consent to participate, depending on their condition.

### Inclusion/Exclusion Criteria

Inclusion criteria were patients 18 years or older, able to provide oral or written consent to participate, and able to understand and express themselves in Norwegian. Exclusion criteria included being in the final stage of a terminal illness.

### Data Collection

Before the study began, all involved nurse anesthetists were informed about the study procedures. Two registration forms were developed based on previous literature (Chopra, 2019; Young & You, 2025) and discussions between the nurse anesthetists responsible for PICC insertion in the hospital: one form (A) for registration of information from patients’ medical records, and one form (B) for registration of information gathered through telephone interviews with patients and searching the medical records longitudinally.

Form A was completed by two specially trained and dedicated nurse anesthetists and included (1) patient characteristics: gender (male, female), hospitalized/outpatient, age (<40 years, 41–50 years, 51–60 years, 61–70 years, 71–80 years, or >80 years), main diagnosis (infection, cancer, dehydration/malnutrition, other), and whether the patient had the PICC when discharged from the hospital (yes, no); (2) suspected length of use of the PICC (<1 week, 1–4 weeks, >4 weeks); (3) insertion procedure: vein and side (basilic, brachial, cephalic, left or right), number of punctures (continuous variable), vein diameter measured by ultrasound without a tourniquet (<4 mm, ≥4 mm, ≥5 mm, ≥6 mm), PICC type (PICC 4Fr one lumen/PICC 4Fr two lumen), and fixation (Statlock, SecurAcath, Statlock with tissue glue, suture, Statlock + SecurAcath, and SecurAcath with tissue glue); (4) PICC indication (antibiotics, nutrition, cytostatics, palliation, fluids, blood sampling, difficult venous access—up to three indications could be registered); and (5) complications registered initially as yes/no. Complications registered included deep venous thrombosis, suspected infection (no bacterial growth on catheter), suspected infection (no test results available), confirmed infection (bacterial growth identified), redness at the insertion point, dislocation of catheter, pain/numbness, and hematoma/bleeding. The length of use of the PICC (<14 days, 14–30 days, >30 days) was calculated when the catheters were discontinued. Form A was completed based on information collected from the patient's medical record.

Form B included (1) patient-reported complications (yes, no). Complications were defined as deep venous thrombosis (verified by a physician/hospital), suspected infection (no bacterial growth on catheter), suspected infection (no test results available), confirmed infection (bacterial growth identified), redness at the insertion point, dislocation of catheter, pain/numbness, and hematoma/bleeding. This information was supplemented with information from searches in the patient's medical record, and (2) if the patient still had the PICC (yes, no; if no: when discontinued). Form B was completed by the two dedicated nurse anesthetists through phone calls to each participating patient approximately every fourth week until the PICC was discontinued (Supplement 2).

Prior to each phone call, the patient's medical record was consulted to assess whether the patient still met the inclusion/exclusion criteria.

### Ethics Approval and Consent to Participate

The project was assessed by the regional ethics committee of South-Eastern Norway (project no. 465247). The study was approved by the Norwegian Knowledge sectors’ service provider (SIKT, project no. 784246), as well as by the hospitals’ GDPR officer. The study was conducted in line with the Declaration of Helsinki (World Medical Assocciation, 2022). All participants provided their informed, willing, and consent to participate. Depending on their condition, patients provided a written consent or an oral consent to participate. Patients were informed that they could withdraw at any point before analysis, without any negative consequences. Many of the patients were severely ill and may be seen as vulnerable. The nurse anesthetists recruiting the patients had extensive experience and were bound by their own professional ethical guidelines. Hence, we assume that no patients were harmed due to participating in the study. In contrast, several of them expressed an appreciation for being contacted repeatedly throughout the period they had the PICC.

### Statistical Analysis

Data were analyzed using the Statistical Package for the Social Sciences version 29. Descriptive statistics and frequencies were used to describe the characteristics of the PICCs and patients, and results are presented as *n* (%). Assessment of normal distribution on the data, using skewness and kurtosis, as well as histograms with linear curve, showed that these were not normally distributed. Chi-square and Fisher's exact test and Spearman's correlations were used as appropriate, to explore covariations between variables. Calculations of missing data were not performed, and missing data are presented as missing, *n* = . *P* values <.05 were considered statistically significant.

## Results

In total, 401 patients were included in the study conducted from August 28, 2022, to April 28, 2023.

### Sample Characteristics

Table 1 provides an overview of the patient and PICC characteristics. All but one of the PICCs were single-lumen, 4Fr.

**Table 1.** Patient and PICC Characteristics (*N* = 401).

	*n* (%)
Male gender	214 (53.4)
Hospitalized	342 (85.3)
PICC at discharge from hospital, yes	328 (83.2)
Age, in years	
18–39	26 (6.5)
40–50	18 (4.5)
51–60	60 (15)
61–70	109 (27.2)
71–80	131 (32.6)
>80	57 (14.2)
Diagnosis	
Infection	224 (55.9)
Cancer	150 (37.4)
Dehydration/malnutrition	17 (4.2)
Other	10 (2.5)
Suspected use (m = 10)	
<1 week	5 (1.3)
1–4 week	190 (48.6)
>4 week	154 (39.4)
Unsure	42 (10.7)
Use of PICC (m = 17)	
<14 days	126 (32.8)
14–30 days	95 (24.7)
>30 days	163 (42.5)
Vein and side (m = 14)	
Basilica, right	267 (69)
Brachialis, right	46 (11.9)
Cephalica, right	2 (0.5)
Basilica, left	65 (16.8)
Brachialis, left	6 (1.5)
Cephalica, left	1 (0.3)
Vein punctions (m = 14)	
1	351 (90.7)
2	27 (7)
3	5 (1.3)
4	4 (1)
Vein diameter (m = 21)	
<4 mm	11 (2.9)
≥4 mm	369 (97.1)
Fixation (m = 4)	
Statlock	127 (32)
Statlock with tissue glue	257 (64.7)
SecurAcath	9 (2.3)
SecurAcath with tissue glue	3 (0.8)
Statlock and SecurAcath	1 (0.2)
Main indication	
Antibiotics	272 (68.3)
Nutrition	46 (11.5)
Cytostatics	42 (10.5)
Palliation	24 (6.1)
Fluids	7 (1.8)
Difficult venous access	7 (1.8)

PICC = peripherally inserted central catheter; Hospitalized = remaining; *n* = outpatients; m = missing; Fr = French.

Table 1 shows that the gender distribution was balanced (53.4% men). Most of the patients were hospitalized (85.3%), and the majority were between 60 and 80 years of age (59.8%). The primary indication for PICC insertion was infection (55.9%), and most PICCs were reported to be needed for less than four weeks (48.6%), even though 42.5% were utilized for more than 30 days. Furthermore, most patients retained their PICC upon discharge (83.2%). The right basilic vein was most frequently used for PICC insertion (69%), with veins at least 4 mm in diameter (97.1%) and primarily after a single vein puncture (90.5%). The primary reason for PICC insertion was the need for antibiotics (68.3%).

### Research Question Results

Table 2 presents the registered PICC complications. Table 2 shows that the most frequent complication registered in the medical records was redness at the insertion point (29.2%), followed by catheter dislocation after four weeks (33.3%). Infection was confirmed in four PICCs (9.8%), accounting for 0.22 infections per 1,000 catheter days.

**Table 2.** Peripherally Inserted Central Catheter Complications.

	*n* (%)
**Complication reported in medical record (*N*** **=** **401), *n*** **=** **41 (10.2%)**	
Redness	12 (29.2)
DVT	5 (12.2)
Hematoma/bleeding	5 (12.2)
Infection, no growth	5 (12.2)
Infection, no test results	4 (9.8)
Infection, confirmed	4 (9.8)
Pain/numbness	4 (9,8)
Dislocation	2 (4.8)
**Complications after 4 weeks (*N*** **=** **297), *n*** **=** **12 (4%)**	
Dislocation	4 (33.3)
Hematoma/bleeding	3 (25)
Infection, no test results	2 (16.7)
Redness	2 (16.7)
Pain/numbness	1 (8.3)

PICC = peripherally inserted central catheter; DVT = deep venous thrombosis; Redness = red at the insertion point.

Infection signs: suspected infection-not bacterial growth on catheter, suspected infection-no test results available, confirmed infection.

Table 3 provides an overview of covariations between complications and factors such as age, duration of PICC use, primary indication for PICC, vein diameter, and number of vein punctures.

**Table 3.** Covariation Between Complication and Potential Risk Factors (*N* = 401).

Age							*p* value
	18–39 *n* (%)	40–50 *n* (%)	51–60 *n* (%)	61–70 *n* (%)	71–80 *n* (%)	>80 *n* (%)	.31^1^
**Complication**	3 (11.5)	1 (5.6)	11 (18.3)	11 (10.1)	11 (8.4)	4 (7)	
**Duration of PICC use**							
	<14 *n* (%)	14–30 *n* (%)	>30 *n* (%)				.44^2^
**Complication**	14 (11.1)	8 (8.4)	15 (9.4)				
**Main indication for PICC (m** **=** **3)**							
	Antibiotics *n* (%)	Nutrition *n* (%)	Cytostatics *n* (%)	Palliation *n* (%)	Fluids *n* (%)		.36^1^
**Complication**	28 (10.3)	3 (6.5)	6 (14.3)	1 (4.2)	2 (28.6)		
**Vein diameter (m** **=** **21)**							
	<4mm	≥4mm < 5mm	≥5mm < 6 mm	≥6mm	Not registered		.70^1^
**Complication**	2 (18.2)	26 (11.5)	9 (9.5)	3 (6.5)	1 (11.1)		

PICC = peripherally inserted central catheter. Complication = yes, as reported in the medical records. Age; in years. Duration of PICC use; in days. Cross-tables.

1 Fisher’s exact test.

2 Kruskal–Wallis test.

*p* < .05 = statistically significant.

As shown in Table 3, none of the covariations were statistically significant. The Spearman's correlation coefficient was 0.03 between complications reported in the medical records and the number of vein punctures, which was not statistically significant (*p* = .63).

## Discussion

This study contributes to the understanding of the characteristics of patients and PICCs inserted in a hospital where nurse anesthetists are primarily responsible for PICC insertion. Of the 401 PICCs included in this study, a total of 53 catheters (13.2%) developed symptoms indicative of a complication or risk of complication.

The main indication for a PICC was the need for IV antibiotics (68.3%), with a reported suspected need for the catheter for less than four weeks (48.6%). Local guidelines state that PICCs should be used if needing IV treatment for more than four weeks, especially if the treatment involves abnormal pH/venous irritants. This suggests that the nurse anesthetists and anesthetists thoroughly assessed the IV access route that seemed most appropriate for the patient. Previous studies on patient experiences with PICCs indicate that patients have positive experiences with these catheters, as they eliminate the need for multiple punctures, catheter changes, and facilitate home treatment (Edström et al., 2016; Leonardsen et al., 2020; Mitchell et al., 2017). Hence, such considerations may have influenced the choice of PICC as the IV route. Swaminathan et al. (2022) longitudinally studied 5,758 patients with PICCs and 5,105 patients with midlines, who received the catheter due to difficult venous access or short-term antibiotic treatment for less than 30 days. Results indicated patients with a midline had lower rates of occlusion (2.1% vs. 7.0%; *p* < .001) and bloodstream infection (0.4% vs. 1.6%; *p* < .001). However, the risk of DVT events was lower in patients who received a PICC compared to a midline (hazard ratio, 0.53; 95% CI, 0.38–0.74). The authors suggested a thoughtful selection between the two catheter types. Therefore, Chopra (2019) specifically recommends using PICCs when patients require tissue-irritating antibiotics.

Both patient and PICC characteristics in the current study were similar to those presented in previous studies, emphasizing older patients, patients with infections or cancer, and those needing antibiotics (Chopra, 2019; Mitchell et al., 2017; Swaminathan et al., 2022). The nurse anesthetists predominantly used veins with a diameter of at least 4 mm in the right upper arm. This is consistent with local guidelines and international recommendations (Chopra, 2019; Nolan et al., 2016). Furthermore, none of the PICCs were sutured in the current study. In a systematic review and meta-analysis, Luo et al. (2017) found a statistically significant association between suturing and catheter-related infections.

In the current study, four patients (1%) developed verified catheter-related infections, as indicated by bacterial growth. This correlates with 0.2 infections per 1,000 catheter days, which is less than the 1.1 reported by Young and You (2025) and 0.46 per 1,000 catheter days by Nolan et al. (2016). However, it is slightly higher than the 0.16 per 1,000 catheter days reported by Taxbro et al. (2019). Moreover, 12 patients (3%) experienced redness, and nine had nonverified (no growth, no test results) symptoms of infection (2.2%) registered in the medical records, while four had nonverified infection/redness (1%) reported by patients. These symptoms may serve as early signs or risks for infection and should have led to close follow-up of the catheter. Nurse anesthetists, who provide information about the PICCs to both hospital wards and municipalities, should include such observations (Leonardsen et al., 2020; Smith & Haraldstad, 2019).

Additionally, five patients (1.2%) developed a DVT. In comparison, Nolan et al. (2016) found a 4% PICC-related DVT incidence, and Taxbro et al. (2019) reported incidences of 16.8% versus 2.1% in vein ports. Paquet et al. (2017) examined the relationship between using the right versus the left upper arm for PICC insertion, concluding that the right arm reduces the risk of DVT, although malignancy was a significant contributor. Moreover, Chopra. (2019) reported a 5.9% incidence of DVTs in cancer patients.

Other identified complications included hematoma/bleeding, pain/numbness, and dislocation, occurring in 19 patients (4.7%). These symptoms may be associated with the insertion procedure. All PICC insertions were ultrasound-guided by trained nurse anesthetists. Heffner and Androes (2024) emphasize the importance of sufficient knowledge and training in ultrasound-guided PICC insertion. We found no statistically significant correlations between complications and patient age, duration of PICC use, primary indication for PICC, vein diameter, or the number of vein punctures. However, most patients in the current study were over 60 years old. Some studies focusing on pediatric patients show an increased risk of catheter-related infections in children under 5 years (Badheka et al., 2019) and in neonates with low gestational age (24–26 weeks), extremely low birth weight, stays for 30 days or more, and older age (aged 10–14 days) (AlGhamdi et al., 2025). We have not identified studies focusing on younger adults and otherwise healthy patients. Therefore, we cannot conclude whether covariations between age and other variables exist in younger, healthier patients.

The PICCs in this study were inserted by nurse anesthetists specifically trained in ultrasound-guided venous access and PICCs. Sakai et al. (2024) found a lower complication rate in PICCs inserted by nurse practitioners compared to physicians (1.5% vs. 5.1%; adjusted odds ratio = 0.31; 95% CI: 0.17–0.59; *p* < .001). Raynak and Wood (2021) found that implementing Vascular Access Clinical Nurse Specialists led to cost reductions by avoiding unnecessary PICC insertions. The advanced role of nurses or nurse anesthetists in venous access or PICC teams warrants further exploration.

### Strengths and Limitations

A strength of this study is the inclusion of 401 PICCs inserted over an 8-month period, followed until they were discontinued. To our knowledge, this is one of the most comprehensive studies of PICCs internationally. However, due to the limited occurrence of complications, further studies with larger samples should be conducted. Also, the PICCs in this study were inserted by nurse anesthetists, and no other studies with similar backgrounds have been identified.

No sample size calculations were performed, and reasons for exclusion or nonparticipation were not systematically registered, which may affect the generalizability of these results to other settings.

The study was conducted in a single-center Norwegian hospital, which might also limit generalizability to different healthcare settings with varying practices. Furthermore, reliance on self-reported complications via patient phone surveys could introduce recall bias or underreporting.

The registration forms were researcher-developed and not validated or psychometrically tested. However, the forms were discussed among nurse anesthetists and anesthetists involved in PICC insertion, enabling claims of face and content validity. A known challenge in documenting catheter-related infections is defining them accurately. In this study, “suspected infection-no bacterial growth on catheter,” “suspected infection-no test results available,” “confirmed infection with bacterial growth detected,” and “redness at the insertion point” were used as definitions of infection. Measures vary between studies, and it may be debated whether including tests such as C-reactive protein, Sed-rate, ultrasound verification of thrombosis, or fever would have offered a more precise identification of infections or other complications.

Time may be a variable increasing the risk of infections. In this study, no time-to-event analysis was performed due to challenges in accurately registering the time of event occurrence. This may be seen as a limitation.

### Implications for Practice

This study indicates that PICC insertion performed by nurse anesthetists is safe and efficient. Complications occur but vary in severity, emphasizing the importance of assessing the pros and cons of such catheters and considering alternatives. Nurse anesthetists must be aware of the potential risks and provide input when deciding on venous access of choice.

## Conclusion

This study contributes valuable insights into the safety and efficacy of nurse anesthetist-led PICC insertions, supporting the expanding role of nurse anesthetists in advanced clinical procedures and identifying areas for further research to optimize patient outcomes. The findings indicate that PICCs are suitable for patients of various ages and diagnoses requiring IV treatment, particularly for long-term treatment. More studies exploring complications versus patient experiences are needed. Additionally, further studies should focus on the use of PICCs in younger patients with few comorbidities requiring long-term IV access.

## Supplemental Material

sj-docx-1-son-10.1177_23779608251367258 - Supplemental material for Nurse Anesthetist-Performed PICC Insertion: A Prospective Longitudinal Study in a Norwegian Hospital Supplemental material, sj-docx-1-son-10.1177_23779608251367258 for Nurse Anesthetist-Performed PICC Insertion: A Prospective Longitudinal Study in a Norwegian Hospital by Ann-Chatrin Linqvist Leonardsen, Ellen Marie Lunde and Mona Sand Andersen in SAGE Open Nursing

sj-docx-2-son-10.1177_23779608251367258 - Supplemental material for Nurse Anesthetist-Performed PICC Insertion: A Prospective Longitudinal Study in a Norwegian Hospital Supplemental material, sj-docx-2-son-10.1177_23779608251367258 for Nurse Anesthetist-Performed PICC Insertion: A Prospective Longitudinal Study in a Norwegian Hospital by Ann-Chatrin Linqvist Leonardsen, Ellen Marie Lunde and Mona Sand Andersen in SAGE Open Nursing
