# Supplementary material for: Nurse Anesthetist-Performed PICC Insertion: A Prospective Longitudinal Study in a Norwegian Hospital
Source: SAGE Open Nurs. 2025 Aug 19;11:23779608251367258. doi: 10.1177/23779608251367258 (PMC12368325; doi:10.1177/23779608251367258)
Supplement: sj-docx-2-son-10.1177_23779608251367258 - Supplemental material for Nurse Anesthetist-Performed PICC Insertion: A Prospective Longitudinal Study in a Norwegian Hospital [file sj-docx-2-son-10.1177_23779608251367258.docx]

**Questionnaire**

1. **Do you still have your PICC? (yes/no)**
2. **Has the PICC been replaced with a new one? (yes/no)**
3. **Is the PICC in use? For what?**
4. **Have you had any PICC related complications (yes/no)**
5. **If yes: which type of complication? (**deep venous thrombosis, suspected infection-not bacterial growth on catheter, suspected infection-no test results available, confirmed infection, red at the insertion point, dislocation of catheter, pain/numbness and hematoma/bleeding)
6. **On a scale from 1-5, where 1=very unsatisfied and 5=very satisfied, how satisfied are you with your PICC?**
7. **Would you choose having a PICC again? (yes/no/unsure)**
8. **Response by (patient/relative/healthcare personnel)**
